# Retraction: Mental health identification of children and young adults in a pandemic using machine learning classifiers

**DOI:** 10.3389/fpsyg.2024.1456387

**Published:** 2024-07-12

**Authors:** Frontiers Editorial Office

Please find the retraction statement below:

Following publication, concerns were raised regarding data misrepresentation. The authors have remained unresponsive to multiple requests for the raw data for this study. Given extant concerns over the validity of the study, the article has been retracted.

This retraction was approved by the Specialty Chief Editor of Educational Psychology and the Editor-in-Chief of Frontiers in Psychology. The authors have not responded to correspondence regarding this retraction.

